# Association of Attention-Deficit/Hyperactivity Disorder Diagnosis With Adolescent Quality of Life

**DOI:** 10.1001/jamanetworkopen.2022.36364

**Published:** 2022-10-13

**Authors:** Luise Kazda, Kevin McGeechan, Katy Bell, Rae Thomas, Alexandra Barratt

**Affiliations:** 1 Sydney School of Public Health, Faculty of Medicine and Health, The University of Sydney, Sydney, Australia; 2 Institute for Evidence-Based Healthcare, Bond University, Robina, Australia

## Abstract

**Importance:**

Appropriate diagnosis of attention-deficit/hyperactivity disorder (ADHD) can improve some short-term outcomes in children and adolescents, but little is known about the association of a diagnosis with their quality of life (QOL).

**Objective:**

To compare QOL in adolescents with and without an ADHD diagnosis.

**Design, Setting, and Participants:**

This cohort study followed an emulated target trial design using prospective, observational data from the Longitudinal Study of Australian Children, a representative, population-based prospective cohort study with biennial data collection from 2006 to 2018 with 8 years of follow-up (ages 6-7 to 14-15 years). Propensity score matching was used to ensure children with and without ADHD diagnosis were well matched on a wide range of variables, including hyperactive/inattentive (H/I) behaviors. Eligible children were born in 1999 to 2000 or 2003 to 2004 and did not have a previous ADHD diagnosis. All incident ADHD cases were matched with controls. Data were analyzed from July 2021 to January 2022.

**Exposures:**

Incident parent-reported ADHD diagnosis at age 6 to 7, 8 to 9, 10 to 11, 12 to 13, or 14 to 15.

**Main Outcomes and Measures:**

Quality of life at age 14 to 15 was measured with Child Health Utility 9D (CHU9D) and 8 other prespecified, self-reported measures mapped to the World Health Organization’s QOL domains. Pooled regression models were fitted for each outcome, with 95% CIs and *P* values calculated using bootstrapping to account for matching and repeat observations.

**Results:**

Of 8643 eligible children, a total of 393 adolescents had an ADHD diagnosis (284 [72.2%] boys; mean [SD] age, 10.03 [0.30] years; mean [SD] H/I Strengths and Difficulties Questionnaire score, 5.05 [2.29]) and were age-, sex-, and H/I score–matched with 393 adolescents without ADHD diagnosis at time zero. Compared with adolescents without diagnosis, those with an ADHD diagnosis reported similar QOL on CHU9D (mean difference, −0.03; 95% CI, −0.07 to 0.01; *P* = .10), general health (mean difference, 0.11; 95% CI, −0.04 to 0.27; *P* = .15), happiness (mean difference, −0.18; 95% CI, −0.37 to 0.00; *P* = .05), and peer trust (mean difference, 0.65; 95% CI, 0.00 to 1.30; *P* = .05). Diagnosed adolescents had worse psychological sense of school membership (mean difference, −2.58; 95% CI, −1.13 to −4.06; *P* < .001), academic self-concept (mean difference, −0.14; 95% CI, −0.02 to −0.26; *P* = .02), and self-efficacy (mean difference, −0.20; 95% CI, −0.05 to −0.33; *P* = .007); displayed more negative social behaviors (mean difference, 1.56; 95% CI, 0.55 to 2.66; *P* = .002); and were more likely to harm themselves (odds ratio, 2.53; 95% CI, 1.49 to 4.37; *P* < .001) than adolescents without diagnosis.

**Conclusions and Relevance:**

In this cohort study, ADHD diagnosis was not associated with any self-reported improvements in adolescents’ QOL compared with adolescents with similar levels of H/I behaviors but no ADHD diagnosis. ADHD diagnosis was associated with worse scores in some outcomes, including significantly increased risk of self-harm. A large, randomized clinical trial with long-term follow-up is needed.

## Key Points

**Question:**

Is an attention-deficit/hyperactivity disorder (ADHD) diagnosis in childhood or early adolescence associated with quality of life in adolescents compared with age-, sex-, and behavior-matched individuals without a diagnosis?

**Findings:**

In this cohort study, 393 adolescents with an ADHD diagnosis reported similar quality of life overall and on 3 subdomains but significantly worse outcomes for 5 other aspects of quality of life compared with 393 matched adolescents with similar levels of hyperactive/inattentive behaviors but no ADHD diagnosis.

**Meaning:**

These findings suggest that childhood ADHD diagnosis may not result in any improvements in quality of life measures in adolescents and may negatively impact some outcomes, such as risk of self-harm.

## Teaser

This cohort study examines quality of life in adolescents who have received a diagnosis of attention-deficit/hyperactivity disorder compared with peers with matched hyperactive/inattentive behaviors but who have not received a diagnosis.

## Introduction

Attention-deficit/hyperactivity disorder (ADHD) is “a persistent pattern of inattention and/or hyperactivity-impulsivity that interferes with functioning…and negatively impacts…social and academic activities”^1^. This negative effect can extend beyond activities directly impacted by hyperactive or inattentive (H/I) behaviors, thus affecting overall quality of life (QOL).^2^ The World Health Organization (WHO) defines QOL as “an individual’s perception of their position in life,”^3^ placing emphasis on the subjective experience and the influence of the environment. Focusing on QOL when measuring impacts of a health condition provides an opportunity to shift away from symptom reduction toward more holistic, patient-centered outcomes.^4^

A 2017 industry-funded, systematic review found some evidence that pharmaceutical treatment of ADHD may be associated with improved QOL in short-term trials; however, this improvement is, at best, much smaller than for symptom reduction.^2^ Other studies found no association with QOL.^5,6^

It is unclear how much of the negative outcomes associated with ADHD in children can be attributed to impairment resulting from the condition itself and how much is due to the labeling effect of the ADHD diagnosis.^7^ H/I behaviors exist on a spectrum and correlate positively with impairment in domains, such as academic performance or socioemotional problems.^8^ Reducing them (eg, through treatment) can lead to some improvements in these domains.^9^ The potential for improvement is smaller for children with fewer H/I behaviors, for whom large reductions are impossible.^10^ For these children, harms associated with the ADHD diagnosis (such as stigma) or from treatment (eg, medication adverse effects) may outweigh the benefits of symptom reduction.^8^

Gender and age are associated with ADHD diagnosis, H/I behaviors, and QOL, with girls being diagnosed with ADHD at much lower rates than boys.^11,12^ Also, some argue that diagnosing (and treating) earlier in life may lead to better outcomes beyond symptom control.^13,14^ In contrast, a watch-and-wait approach favoring later diagnosis may avoid potential harms.^15^

The rationale for this study was to estimate the association of ADHD diagnosis with self-perceived QOL in adolescents. An ADHD diagnosis usually triggers intervention, either pharmacological or nonpharmacological, which should benefit the individual.^16^ We hypothesized that children and adolescents benefit from an ADHD diagnosis and that this may result in better QOL among adolescents with diagnosis compared with youths with similar H/I behaviors and demographic characteristics but without ADHD diagnosis.

We aimed to investigate whether an ADHD diagnosis in childhood or early adolescence is associated with improved QOL in adolescents compared with well-matched individuals without diagnosis. Additionally, we examined if any association of an ADHD diagnosis with QOL differed by sex, degree of H/I behaviors, or age at first diagnosis.

## Methods

This study was approved by The University of Sydney’s Human Ethics Research Committee. Informed consent was obtained when the children were recruited into the original cohort study. This study was conducted as a secondary analysis and did not directly recruit any participants. We followed the Strengthening the Reporting of Observational Studies in Epidemiology (STROBE) reporting guideline.

### Approach and Study Design

We used a target trial approach to conceptualize an ideal pragmatic trial (as described by Hernán^17^: “one in which treatment strategies are compared under the usual conditions in which they will be applied”) and then emulated it^18^ as closely as possible, given the available observational data (eTable 1 in the Supplement). We used a new-user design to avoid confounding by diagnosis and/or treatment, mimicking random assignment to either the exposure (ADHD diagnosis) or control (no diagnosis) group at each age (6-7, 8-9, 10-11, 12-13, and 14-15 years). Thus, 5 nested, emulated trials were created, each with different age of first diagnosis (the time zero when follow-up starts).^17^ For each, we used propensity scores to select controls who were most similar to children in the exposure group on a number of measured potential confounders. Eligible children were able to be included multiple times. Propensity scores were calculated at each of the 5 time-zeros, based on data collected at the previous wave, to ensure that covariates were unaffected by emulated assignment to either group. Children in the exposure group and their matched controls were then followed from time zero until age 14 to 15 years, when outcomes were measured. Thus, length of follow-up varied between 8 years (time zero at age 6-7 years) and no years (time zero at age 14-15 years). As the contrast of interest^18^ was the observational analogue of an intention-to-treat effect, we did not account for crossover during follow-up (but we report it).

### Setting and Participants

We used data from the Longitudinal Study of Australian Children (LSAC), a representative, population-based sample. A total of 4983 children were recruited into cohort 1 (born 1999-2000) and 5107 children into cohort 2 (born 2003-2004). Full details are published elsewhere.^19^ We combined data from both cohorts and used 5 biennial waves of data collection, in which children were aged 6 to 7, 8 to 9, 10 to 11, 12 to 13, and 14 to 15 years (cohort 1: 2006-2014; cohort 2: 2010-2018). We included data at age 4 to 5 years to determine preselection demographics for age 6 to 7 years.

### Eligibility Criteria

We restricted eligibility to children between the ages of 6 and 15, for whom data were available at age 6 to 7 years and with no reported ADHD diagnosis prior to age 6 years. At each subsequent wave, children either entered the exposure group if a new ADHD diagnosis was reported or the control group if they remained undiagnosed and were matched to a child in the exposure group (Figure).

**Figure.**
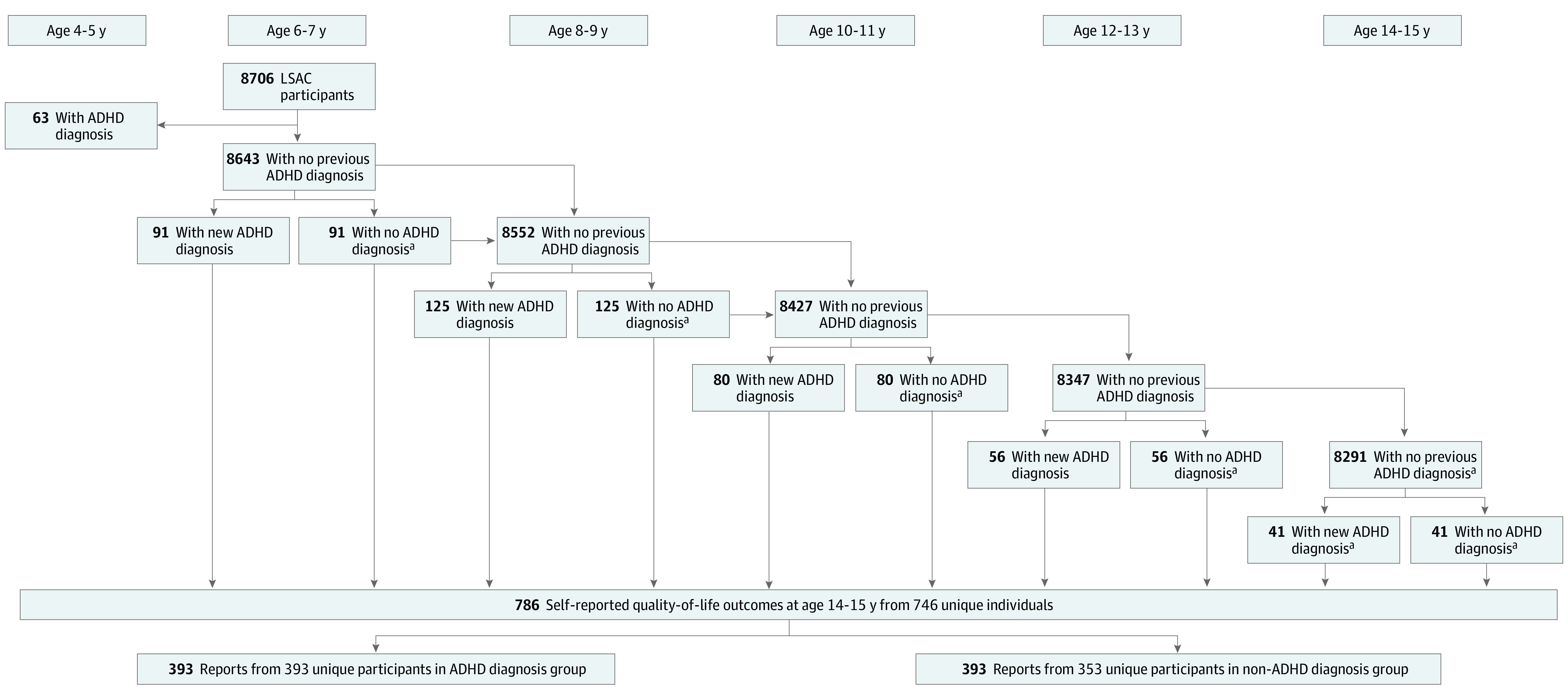
Participant Selection Flowchart ^a^Propensity score–matched controls. Data were calculated from the mean of 25 multiple imputation sets. ADHD indicates attention-deficit/hyperactivity disorder; LSAC, Longitudinal Study of Australian Children.

### Exposure and Control Groups and Follow-up

At each wave, children whose parents answered yes to the question “Does the study child have attention-deficit disorder or attention-deficit/ hyperactivity disorder?” for the first time were assigned to the exposure group. We used an optimal matching propensity score approach to select controls without a parent-reported ADHD diagnosis and paired them in a 1:1 ratio. We forced exact matching on sex to ensure equal numbers of boys and girls in each group.

Importantly, we included a measure of H/I behaviors in the propensity score, using the parent-reported H/I subscale of the Strengths and Difficulties Questionnaire (SDQ).^20^ This validated screening instrument combines 5 Likert-scale items to create a score between 0 and 10, with higher scores signaling increasing risk of clinical ADHD.^20^

Other variables in the propensity score were LSAC birth cohort, Aboriginal and/or Torres Strait Islander status, household features (main language spoken at home, 2-parent household, number of siblings, stressful life events) location (rurality and state of residency), socioeconomic indicators (Socio-Economic Indexes for Areas^21^ advantage or disadvantage score, socioeconomic position *z*-score,^22^ mother’s education), and comorbidities (depression or anxiety, autism, and any physical comorbidity). Aboriginal and/or Torres Strait Islander status was determined by asking parents the question “Is Study Child of Aboriginal or Torres Strait Islander origin?” with answer options no; yes, Aboriginal; yes, Torres Strait Islander; and yes, both. Children whose parents answered anything but no were determined to be of Aboriginal and/or Torres Strait Islander origin. Australian Indigenous children generally have worse health outcomes than non-Indigenous children, thus this was treated as a potential confounder for this analysis. The Socio-economic Advantage and Disadvantage score^21^ summarizes information about the economic and social conditions within an area, with lower scores indicating greater disadvantage and less advantage (at age 6-7 years, the range within the LSAC study was 649.64-1207.26). The socioeconomic position *z*-score is a combination measure developed by LSAC researchers as an indicator of socioeconomic status.^22^ It measures parental education level, income, and occupational status and ranks each family in terms of their socioeconomic position relative to all other families in the sample,^22^ with higher values indicating relatively higher socioeconomic position (at age 6-7 years, the range within the LSAC study was −6.95 to 3.20).We conducted multiple imputations with fully conditional methods, creating 25 complete data sets with imputed values for all dependent and independent variables to account for missing data.

### Outcomes

Our outcome measures were chosen to cover all aspects of the WHO domains of QOL^23^ and are all self-reported measures to focus on adolescents’ own perceptions. We predetermined the validated Child Health Utility 9D (CHU9D) as the primary outcome measure because it comprehensively measures QOL across 9 domains and is a preference-based tool, which has been found to be robust in both children and adolescents.^24,25^ Secondary outcome measures included academic self-concept, global health, negative social behaviors, overall happiness, peer trust, psychological sense of school membership, self-efficacy, and self-harm (eTable 2 in the Supplement).

### Statistical Analysis

We first created 25 complete data sets using multiple imputation, then created 5 emulated trials (through propensity score matching at each age point) for each data set. We then pooled data across all emulated trials for all imputed data sets. We conducted linear regression analysis to estimate mean differences for 8 continuous outcomes and logistic regression to estimate an odds ratio for 1 binary outcome, including age at time zero as a design variable. To investigate potential modification, we constructed models that separately included interaction terms between ADHD diagnosis and H/I SDQ score, sex, and age at time zero. For this, we categorized the H/I SDQ variable into low (0-4), borderline (5-7), and high-risk (8-10) bands for clinically significant ADHD.^26,27^ To compute the 95% CIs and *P* values accounting for increased uncertainty due to multiple imputations and repeated measures, we used bootstrapping based on 500 paired-level resamplings. *P* values were 2-sided, and statistical significance was set at *P* = .05. Additionally, to assess effect sizes, we calculated Cohen *d* using the root mean square error to approximate the SD. We ran a complete-case sensitivity analysis. We conducted all analyses using SAS statistical software version 9.4M6 STAT 15.1 (SAS Institute). Data were analyzed from July 2021 to January 2022.

## Results

### Baseline Characteristics and Matching

A total of 393 adolescents with an ADHD diagnosis (284 [72.2%] boys; mean [SD] age, 10.03 [0.30] years; mean [SD] H/I Strengths and Difficulties Questionnaire score, 5.05 [2.29]) were age-, sex-, and H/I score–matched with 393 adolescents without ADHD diagnosis (Table 1). Propensity score–matched exposure and control groups were very similar on all potentially confounding variables with no substantial differences between groups at any age or between pooled groups of all 5 emulated trials. Since we forced exact matching on sex, the groups contained equal numbers of boys and girls (Table 1).

**Table 1.** Baseline Characteristics at Time Zero^a^

Characteristic	Participants by ADHD diagnosis, No. (%)														Difference, % (95% CI)
	Age 6-7 y		Age 8-9 y		Age 10-11 y		Age 12-13 y			Age 14-15 y			Pooled (all ages)		
	No (n = 91)	Yes (n = 91)	No (n = 125)	Yes (n = 125)	No (n = 80)	Yes (n = 80)	No (n = 56)	Yes (n = 56)	No (n = 41		Yes (n = 41)	No (n = 393)		Yes (n = 393)	
**Cohort**															
Cohort 1	52 (57.1)	53 (58.2)	72 (57.6)	74 (59.2)	45 (56.2)	46 (57.5)	29 (51.8)	28 (50.0)	21 (51.2)		21 (51.2)	219 (55.7)		222 (56.5)	−0.8 (−7.7 to 6.2)
Cohort 2	39 (42.9)	38 (41.8)	53 (42.4)	51 (40.8)	35 (43.8)	34 (42.5)	27 (48.2)	28 (50.0)	20 (48.8)		20 (48.8)	174 (44.3)		171 (43.5)	0.8 (−6.2 to 7.7)
**Participant**															
Sex^b^															
Boys	65 (71.4)	65 (71.4)	96 (76.8)	96 (76.8)	63 (78.8)	63 (78.8)	36 (64.3)	36 (64.3)	24 (58.5)		24 (58.5)	284 (72.3)		284 (72.3)	NA
Girls	26 (28.6)	26 (28.6)	29 (23.2)	29 (23.2)	17 (21.3)	17 (21.3)	20 (35.7)	20 (35.7)	17 (41.5)		17 (41.5)	109 (27.7)		109 (27.7)	NA
Age at data collection, mean (SD), y	6.86 (0.28)	6.91 (0.28)	8.88 (0.29)	8.88 (0.27)	10.90 (0.32)	10.87 (0.33)	12.96 (0.34)	12.94 (0.32)	14.92 (0.37)		14.87 (0.36)	10.03 (0.31)		10.03 (0.30)	0.0 (−0.04 to 0.04)
Aboriginal or Torres Strait Islander status^c^															
Yes	5 (5.5)	4 (4.4)	7 (5.6)	9 (7.2)	4 (5.0)	5 (6.3)	3 (5.4)	3 (5.4)	1 (2.4)		1 (2.4)	20 (5.1)		22 (5.6)	−0.5 (−3.7 to 2.6)
No	86 (94.5)	87 (95.6)	118 (94.4)	116 (92.8)	76 (95.0)	75 (93.7)	53 (94.6)	53 (94.6)	40 (97.6)		40 (97.6)	373 (94.9)		371 (94.4)	0.5 (−2.6 to 3.7)
**Household**															
English is main language	88 (96.7)	89 (97.8)	115 (92.0)	115 (92.0)	74 (92.5)	74 (92.5)	52 (92.9)	53 (94.6)	39 (95.1)		39 (95.1)	368 (93.6)		370 (94.1)	−0.5 (−3.9 to 2.8)
2 Parents in the home	74 (81.3)	74 (81.3)	99 (79.2)	100 (80.0)	65 (81.3)	65 (81.3)	47 (83.9)	47 (83.9)	36 (87.8)		35 (85.4)	321 (81.7)		321 (81.7)	0.0 (−5.4 to 5.4)
Siblings, mean (SD), No.	1.05 (1.48)	1.15 (1.45)	0.98 (1.48)	0.95 (1.50)	1.06 (1.63)	1.04 (1.61)	1.21 (1.67)	1.17 (1.66)	1.35 (1.83)		1.13 (1.73)	1.08 (1.57)		1.06 (1.56)	0.0 (−0.1 to 0.2)
Stressful life events, mean (SD), No.	1.74 (1.54)	1.75 (1.54)	2.60 (2.74)	2.53 (2.82)	1.99 (1.92)	1.98 (1.95)	2.53 (2.77)	1.93 (2.77)	2.51 (2.77)		3.25 (2.52)	2.26 (2.30)		2.23 (2.31)	0.0 (−0.3 to 0.3)
Rurality															
Major city	62 (68.1)	61 (67.0)	83 (66.4)	84 (67.2)	46 (57.5)	46 (57.5)	31 (55.4)	31 (55.4)	31 (75.6)		31 (75.6)	253 (64.4)		253 (64.4)	0.0 (−6.7 to 6.7)
Rural and remote	29 (31.9)	30 (33.0)	42 (33.6)	41 (32.8)	34 (42.5)	34 (42.5)	25 (44.6)	25 (44.6)	10 (24.4)		10 (24.4)	140 (35.6)		140 (35.6)	0.0 (−6.7 to 6.7)
State of residency															
New South Wales and Australian Capital Territory	39 (42.9)	40 (44.0)	51 (40.8)	52 (41.6)	35 (43.8)	34 (42.5)	22 (39.3)	22 (39.3)	13 (31.7)		13 (31.7)	160 (40.7)		161 (41.0)	−0.3 (−7.1 to 6.6)
Victoria and Tasmania	13 (14.3)	14 (15.4)	20 (16.0)	20 (16.0)	10 (12.5)	9 (11.3)	15 (26.8)	15 (26.8)	10 (24.4)		9 (22.0)	68 (17.3)		67 (17.0)	0.3 (−5.0 to 5.5)
Queensland and Northern Territory	24 (26.4)	22 (2.2)	43 (34.4)	42 (33.6)	21 (26.3)	23 (28.8)	13 (23.2)	12 (21.4)	14 (34.1)		14 (34.1)	115 (29.3)		113 (28.8)	0.5 (−5.8 to 6.9)
South Australia and Western Australia	15 (16.5)	15 (16.5)	10 (8.0)	11 (8.8)	14 (17.5)	14 (17.5)	7 (12.5)	7 (12.5)	4 (9.8)		5 (12.2)	50 (12.7)		52 (13.2)	0.5 (−5.2 to 4.2)
**Socioeconomic indicators, mean (SD)**															
SEIFA advantage/disadvantage score	1000.72 (74.89)	999.73 (71.63)	1002.29 (74.14)	1004.37 (72.84)	993.07 (74.83)	992.90 (81.04)	1015.29 (78.25)	1013.83 (70.63)	1001.32 (81.21)		1009.19 (88.93)	1001.80 (75.78)		1002.81 (75.59)	−1.0 (−11.6 to 9.6)
Socioeconomic position, *z*-score (SD)	−0.26 (0.90)	−0.27 (1.09)	−0.15 (0.98)	−0.15 (0.97)	−0.26 (0.93)	−0.27 (0.90)	0.04 (1.00)	0.06 (0.93)	0.04 (0.96)		0.09 (1.17)	−0.15 (0.95)		−0.15 (1.00)	0.00 (−0.1 to 0.1)
Mother’s school completion															
≤Year 11	41 (45.1)	41 (45.1)	54 (43.2)	55 (44.0)	30 (37.5)	31 (38.8)	18 (32.1)	19 (33.9)	16 (39.0)		15 (36.6)	159 (40.5)		161 (41.0)	−0.5 (−7.4 to 6.4)
Year 12 or equivalent	50 (54.9)	50 (54.9)	71 (56.8)	70 (56.0)	50 (62.5)	49 (61.3)	38 (67.9)	37 (66.1)	25 (61.0)		26 (63.4)	234 (59.5)		232 (59.0)	0.5 (−6.4 to 7.4)
Mother’s further education															
None	32 (35.2)	34 (38.5)	33 (26.4	31 (24.8)	21 (26.3)	16 (20.0)	10 (17.9)	9 (16.1)	8 (19.5)		6 (14.6)	104 (26.5)		96 (24.4)	2.0 (−4.1 to 8.1)
Vocational or other	40 (44.0)	35 (38.5)	55 (44.0)	57 (45.6)	37 (46.3)	47 (58.8)	25 (44.6)	26 (46.4)	20 (48.8)		21 (51.2)	177 (45.0)		186 (47.3)	−2.3 (−9.3 to 4.7)
University degree	20 (22.0)	22 (24.2)	37 (29.6)	36 (28.8)	22 (27.5)	17 (21.3)	21 (37.5)	21 (37.5)	14 (34.1)		14 (34.)	114 (29.0)		110 (28.0)	1.0 (−5.3 to 7.3)
**Comorbidities^d^**															
Depressionor anxiety	NA	NA	NA	NA	NA	NA	(7) 12.5	(8) 14.3	(5) 12.2		(7) 17.1	(12) 12.		(15) 15.5	−3.1 (−12.8 to 6.6)
Autism	NA	NA	NA	NA	NA	NA	(6) 10.7	(8) 14.3	(2) 4.9		(2) 4.9	(8) 8.2		(10) 10.3	−2.1 (−10.2 to 6.1)
Other comorbidities, mean (SD), No.	0.49 (0.76)	0.49 (0.66)	0.44 (0.70)	0.44 (0.65)	0.82 (1.05)	0.76 (0.88)	0.93 (1.27)	1.00 (1.21)	0.71 (1.03)		0.68 (1.05)	0.63 (0.90)		0.62 (0.82)	0.0 (−0.1 to 0.1)
SDQ H/I scores															
Mean (SD) score	5.69 (2.24)	5.65 (2.29)	5.19 (2.31)	5.50 (2.18)	4.93 (2.31)	5.14 (2.20)	4.43 (2.30)	4.55 (2.34)	4.24 (2.25)		3.77 (2.26)	5.05 (2.29)		5.15 (2.24)	−0.1 (−0.4 to 0.2)
Low risk (0-4)	26 (28.6)	29 (31.9)	49 (39.2)	41 (32.8)	36 (45.0)	30 (37.5)	29 (51.8)	27 (48.2)	23 (56.1)		26 (63.4)	163 (41.5)		153 (38.9)	2.5 (−4.3 to 9.4)
Borderline (5-7)	44 (48.4)	40 (44.0)	55 (44.0)	61 (48.8)	31 (38.8)	37 (46.3)	20 (35.7)	21 (37.5)	14 (34.1)		11 (26.8)	164 (41.7)		170 (43.3)	−1.5 (−8.4 to 5.4)
High risk (8-10)	21 (23.1)	22 (24.2)	22 (17.6)	23 (18.4)	13 (16.3)	12 (15.0)	6 (10.7)	8 (14.3)	4 (9.8)		4 (9.8)	66 (16.8)		69 (17.6)	−0.8 (−6.0 to 4.5)

Abbreviations: ADHD, attention-deficit/hyperactivity disorder; SDQ H/I, Strengths and Difficulties Questionnaire, Hyperactive/Inattentive Score; SEIFA, Socio-Economic Indexes for Areas.

^a^ Mean from 25 multiple imputation sets.

^b^ Based on parent-reported biological sex at age 6 to 7 years, not gender, with boy or girl the 2 only options available.

^c^ Aboriginal and/or Torres Strait Islander status was determined by asking parents the question “Is Study Child of Aboriginal or Torres Strait Islander origin?” with answer options no; yes, Aboriginal; yes, Torres Strait Islander; and yes, both. Children whose parents answered anything but no were determined to be of Aboriginal and/or Torres Strait Islander origin.

^d^ Not all parents were asked about child’s depression or anxiety and autism before age 12/13 years.

This balance is reflected in the standardized mean differences (SMD) and variance ratios of the propensity scores and of the individual variables (eTable 3 in the Supplement). SMDs and variance ratios for all propensity scores remained well below the recommended thresholds for adequate balance (SMD threshold, 0.1; variance ratio threshold: 2.0).^28^ Autism and depression or anxiety were above this threshold at 1 age each (eTable 3 in the Supplement), most likely owing to very small numbers of individuals affected (6 vs 8 adolescents with autism at age 12 to 13 years and 5 vs 7 adolescents with depression or anxiety at age 14 to 15 years).

### Attrition, Missing Data, and Adherence

Data were available for 8643 children without a previous ADHD diagnosis at age 6 to 7 years. Of these, 6433 adolescents (74.6%) remained in the study at age 14 to 15 years, with some missing data for several variables at each age. We imputed data for all missing values (dependent and independent variables) and for participants lost to follow-up. Thus, we used data from all participants who were present at age 6 to 7 years, creating data sets with the same number of participants to select from at every age (8643 participants) (eTable 4 in the Supplement). We report on drop-ins and drop-outs for both groups (eTable 5 in the Supplement), showing that 273 adolescents (69.6%) in the exposure group still recorded an ADHD diagnosis at the end of follow-up while 18 participants (4.6%) in the control group received the diagnosis at some point during follow-up. We ran a complete-case analysis as a sensitivity analysis with data from 5535 children who remained in the study at age 14 to 15 years and had complete data on all outcomes and covariates.

### Main Analysis

In pooled regression analysis comparing all 5 exposure groups with all controls, we found no difference in QOL, measured by the CHU9D (mean difference, −0.03; 95% CI, −0.07 to 0.01; *P* = .10). There was no difference between groups in perceived health (mean difference, 0.11; 95% CI, −0.04 to 0.27; *P* = .15), happiness (mean difference, −0.18; 95% CI, −0.37 to 0.00; *P* = .05), or peer trust (mean difference, 0.65; 95% CI, 0.00 to 1.30; *P* = .05). On 4 other measures, adolescents with ADHD diagnosis reported worse outcomes than those without: they had lower academic self-concepts (mean difference, −0.14; 95% CI, −0.26 to −0.02; *P* = .02), more negative social behaviors (mean difference, 1.56; 95% CI, 0.55 to 2.66; *P* = .002), a worse sense of school membership (mean difference, −2.58; 95% CI, −4.06 to −1.13; *P* < .001), and less self-efficacy (mean difference, −0.20; 95% CI, −0.33 to −0.05). The effect sizes, measured by Cohen *d*, for these 4 outcomes were all small (Table 2). Youths with ADHD diagnosis had substantially higher odds for self-harm compared with those without diagnosis (odds ratio, 2.53, 95% CI, 1.49-4.37).

**Table 2.** Association of ADHD Diagnosis With Quality-of-Life Outcomes at Age 14/15 Years

Outcome	ADHD diagnosis, mean (SD)		Mean difference (95% CI)	*P* value	Effect size^a^
	Without (n = 393)	With (n = 393)			
Child Health Utility 9D^b^	0.80 (0.21)	0.77 (0.23)	−0.03 (−0.07 to 0.01)	.10	0.14
Academic self-concept^c^	2.84 (0.66)	2.70 (0.66)	−0.14 (−0.26 to −0.02)	.02	0.21
Global health^d^	2.18 (0.88)	2.30 (0.92)	0.11 (−0.04 to 0.27)	.15	0.12
Negative social behaviors^e^	2.01 (5.04)	3.58 (6.81)	1.56 (0.55 to 2.66)	.002	0.26
Overall happiness^f^	3.77 (1.12)	3.58 (1.13)	−0.18 (−0.37 to 0.00)	.05	0.16
Peer trust^g^	9.62 (3.82)	10.27 (4.08)	0.65 (0.00 to 1.30)	.05	0.16
Psychological sense of school membership^h^	46.86 (8.22)	44.28 (9.05)	−2.58 (−4.06 to −1.13)	<.001	0.30
Self-efficacy^i^	3.85 (0.76)	3.65 (0.83)	−0.20 (−0.33 to −0.05)	.007	0.25
Self-harm, No. %	36 (9.2)	79 (20.2)	2.53 (1.49 to 4.37)^j^	<.001	NA

Abbreviations: ADHD, attention-deficit/hyperactivity disorder; NA, not applicable.

^a^ Cohen *d* were categorized as less than 0.20, negligible effect; 0.20 to 0.49, small effect; 0.50 to 0.79, moderate effect; and 0.80 or greater, large effect.

^b^ Range, 0 to 1; higher scores indicate better quality of life.

^c^ Range, 1 to 4; higher scores indicate a more positive academic self-concept.

^d^ Range, 1 to 5; lower scores indicate better health perception.

^e^ Range, 0 to 85; lower scores indicate fewer negative social behaviors.

^f^ Range, 1 to 5; higher scores indicate more happiness.

^g^ Range, 4 to 20; lower scores indicate better peer trust.

^h^ Range, 12 to 60; higher scores indicate better sense of membership.

^i^ Range, 1 to 5; higher scores indicate better self-efficacy.

^j^ Expressed as an odds ratio (95% CI).

Results from the complete-case sensitivity analysis were unchanged with the exception of peer trust, which crossed the threshold for statistical significance to become significantly worse in diagnosed adolescents in this analysis (mean difference, 0.86; 95% CI, 0.15-1.61) (eTable 6 in the Supplement).

The associations of ADHD diagnosis with QOL outcomes were similar across all H/I SDQ score bands (eTable 7 in the Supplement). Nevertheless, differences between exposure and control groups were generally smaller among participants with the most H/I behaviors, as these adolescents (diagnosed and undiagnosed) reported more unfavorable outcomes than adolescents with fewer H/I behaviors. We found no evidence for modification by sex, with ADHD diagnosis having a comparable association with QOL in girls as in boys (eTable 8 in the Supplement). However, girls reported poorer QOL outcomes than boys, regardless of diagnosis. Happiness and peer trust were better in children first diagnosed at age 14 to 15 years compared with controls, and self-harm was lower in children first diagnosed at age 12 to 13 years, although these differences were not statistically significant. A pattern indicated that the largest (unfavorable) associations of diagnosis with QOL outcomes generally occurred in those first diagnosed at age 6 to 7 years (eTable 9 in the Supplement).

## Discussion

In this cohort study, we found that children diagnosed with ADHD reported similar or poorer QOL at age 14 to 15 years compared with children who had grown up experiencing the same levels of H/I behaviors but had not been given an ADHD diagnosis. We did not observe any differences between groups for overall QOL measured by the CHU9D, perceived health, happiness, or trust. ADHD diagnosis had significant negative associations with academic self-concept, negative social behaviors, sense of school membership, self-efficacy, and self-harm. Our findings were generally applicable to boys and girls. Negative associations with diagnosis were smaller in adolescents with the most H/I behaviors and largest in adolescents diagnosed earliest (age 6-7 years). In summary, among matched adolescents, an ADHD diagnosis was not associated with improved QOL but did have some negative associations, including an increase in risk of self-harm.

### In Context

Our results confirm those reported in other studies, finding that QOL is often lower in adolescents with ADHD compared with those without.^4^ However, to our knowledge, our study is the first that explicitly examined the association of the diagnosis with QOL. Other studies in this field have typically used clinically referred samples in which symptomatic participants with documented diagnoses are compared with controls without diagnosis and without symptoms, making it impossible to disentangle the effects of the disorder itself from those of the intervention in QOL.^4,6^ Previous intervention studies in this field have focused on medication rather than diagnosis, consequently only including participants with clinical ADHD diagnosis.^2,5^ Thus, our study expands the current knowledge that children with ADHD often experience reduced QOL by showing that at least some of this is associated with the diagnosis itself.

While most studies in this field used parent-reported QOL, our findings align well with those that found smaller to no associations of ADHD with self-reported QOL measures.^2,29^ Interestingly, parents of children with health issues tend to rate their children’s QOL lower than the children do themselves, while healthy children rate it lower than their parents do.^29^ Although this issue is beyond the scope of this study, we specifically designed our research to only include self-reported outcome measures to avoid bias due to parent reporting.

Research on the impact of diagnostic labels in other conditions has found beneficial and harmful associations in several areas, such as psychosocial impact, support, future planning, behavior, and treatment expectations.^7^ This is reflected in ADHD studies that have reported positive outcomes associated with diagnoses by creating a sense of empowerment and enablement as well as negative associations through disempowerment and stigma.^8^ We are not aware of research specifically on the association of the ADHD diagnostic label with QOL.

A diagnosis is usually a prerequisite for treatment, especially for pharmaceutical interventions. While there is limited evidence that ADHD medication increases QOL, research supports a general benefit of treatment on numerous related domains.^9^ However, it is also known that medication comes with numerous harms that may outweigh benefits, especially when combined with harms from the diagnosis and in children without major impairment, ie, those with few or borderline H/I behaviors.^30,31^ Still, studies suggest that even in children with subthreshold symptoms, impairment can occur, and they could benefit from additional support.^32,33^

Unfortunately, our results indicate no beneficial associations of an ADHD diagnosis with adolescents’ QOL, which is highly concerning. It implies that the harms associated with an ADHD label (such as stigma, prejudice, deflection from other problems, or the perceived inability to change^8^) may not be offset by benefits associated with the diagnosis or treatment. This is problematic, as it indicates that youths may be harmed by the diagnosis and that interventions to support them are not achieving the desired effect.

### Implications

Our findings highlight the critical need for a large, well-designed, pragmatic trial to evaluate the long-term impact of an ADHD diagnosis on children’s and adolescents’ overall well-being. Moreover, accompanying qualitative research is also urgently needed to further investigate the lived experience of youths, especially their perceptions of benefits and harms associated with an ADHD diagnosis.

These results also emphasize the requirement for a cautious clinical approach to ADHD diagnosis in children and adolescents, while underscoring the need for more targeted support to increase QOL in girls and in children with high-risk levels of H/I behaviors, whether diagnosed or not. Policy makers, researchers, and clinicians are urged to work together to develop more systemic solutions to overcome inequities in education and other settings that disadvantage youths because of their gender or neurodiversity, rather than solely relying on medicalizing and labeling them.

### Strengths and Limitations

A strength of our study is the explicit use of the target trial approach.^18^ The creation of 5 nested, emulated trials enabled us to avert selection bias and minimize confounding, increasing our confidence in the results. Moreover, we were able to draw on a large data set from a representative, population-based cohort study, with data from 8643 children covering 10 years, making our study results generalizable to the Australian population more broadly. The richness of the data set permitted us to closely match controls on an extensive range of covariates, minimizing confounding. Using multiple imputations enabled us to overcome problems with selection bias often inherent in longitudinal studies. Furthermore, we predetermined the exclusive use of self-reported outcome measures instead of parent-reported proxy measures, allowing us to capture the lived experience of adolescents themselves. Also, we specifically selected validated instruments to measure outcomes across all QOL aspects.

Our study has some limitations. Importantly, while our approach emulates a target trial, we base our findings on observational, not randomized clinical trial data. As is the case with any observational analysis, we cannot be certain that our results are not influenced by residual confounding. We attempted to incorporate measures of all potential confounders into our propensity scores but were only able to rely on 1 proxy measure of H/I behaviors. A further limitation is study size: although the original data set was very large, the subset of youths with ADHD diagnosis and matched controls only incorporated 746 individuals. Especially for our modification subanalyses, this sample size may have been too small to detect differences.

## Conclusions

This cohort study found that adolescents with a prior ADHD diagnosis did not report better QOL at age 14 to 15 years compared with matched adolescents without diagnosis and with similar H/I behaviors. This raises important questions about the long-term effectiveness and safety of diagnosing children and adolescents with ADHD, especially for those with low-risk or borderline H/I behaviors. Our findings provide a rationale for a well-designed, large, randomized trial with long-term follow-up.

## Notes

Supplement. **eTable 1.** Target Trial Protocol: Specification and Emulation Using Observational Data **eTable 2.** Outcome Measures **eTable 3.** Propensity Score Balance Assessment **eTable 4. **Missing Data **eTable 5.** Number of Participants in Exposure (Drop-outs) and Control (Drop-ins) Groups with an ADHD Diagnosis During Follow-up **eTable 6.** Association of ADHD Diagnosis With Quality-of-Life Outcomes at Age 14/15 Years: Complete Case Analysis **eTable 7.** Association of ADHD Diagnosis With Quality of Life by H/I SDQ Score **eTable 8.** Association of ADHD Diagnosis With Quality of Life by Sex **eTable 9.** Association of ADHD Diagnosis With Quality of Life at Age 14/15 Years by Age at First Diagnosis (Time Zero)
