# Supplementary material for: Association of Attention-Deficit/Hyperactivity Disorder Diagnosis With Adolescent Quality of Life
Source: JAMA Netw Open. 2022 Oct 13;5(10):e2236364. doi: 10.1001/jamanetworkopen.2022.36364 (PMC9561944; doi:10.1001/jamanetworkopen.2022.36364)
Supplement: Supplement. — eTable 1. Target Trial Protocol: Specification and Emulation Using Observational Data eTable 2. Outcome Measures eTable 3. Propensity Score Balance Assessment eTable 4. Missing Data eTable 5. Number of Participants in Exposure (Drop-outs) and Control (Drop-ins) Groups with an ADHD Diagnosis During Follow-up eTable 6. Association of ADHD Diagnosis With Quality-of-Life Outcomes at Age 14/15 Years: Complete Case Analysis eTable 7. Association of ADHD Diagnosis With Quality of Life by H/I SDQ Score eTable 8. Association of ADHD Diagnosis With Quality of Life by Sex eTable 9. Association of ADHD Diagnosis With Quality of Life at Age 14/15 Years by Age at First Diagnosis (Time Zero) [file jamanetwopen-e2236364-s001.pdf]

Supplemental Online Content

Kazda L, McGeechan K, Bell K, Thomas R, Barratt A. Association of attention-deficit/hyperactivity disorder diagnosis with adolescent quality of life. *JAMA Netw Open*. 2022;5(10):e2236364. doi:10.1001/jamanetworkopen.2022.36364

eTable 1. Target Trial Protocol: Specification and Emulation Using Observational Data

eTable 2. Outcome Measures

eTable 3. Propensity Score Balance Assessment

eTable 4. Missing Data

eTable 5. Number of Participants in Exposure (Drop-outs) and Control (Drop-ins) Groups with an ADHD Diagnosis During Follow-up

eTable 6. Association of ADHD Diagnosis With Quality-of-Life Outcomes at Age 14/15 Years: Complete Case Analysis

eTable 7. Association of ADHD Diagnosis With Quality of Life by H/I SDQ Score

eTable 8. Association of ADHD Diagnosis With Quality of Life by Sex

eTable 9. Association of ADHD Diagnosis With Quality of Life at Age 14/15 Years by Age at First Diagnosis (Time Zero)

This supplemental material has been provided by the authors to give readers additional information about their work.

© 2022 Kazda L et al. *JAMA Network Open.*

eTable 1. Target Trial Protocol: Specification and Emulation Using Observational Data[a]

**Protocol component**	**Description**	**Target trial specification**	**Emulation using LSAC data**
Eligibility criteria	Who will be included in the study?	Children between the ages of 6 and 15 with no prior parent-report of ADHD diagnosis	Analysis is restricted to children who meet the eligibility criteria of the target trial<br>Required data for each person: age, history of ADHD diagnosis
Intervention strategy	What interventions will eligible persons receive?	1. ADHD diagnosis<br>2. No ADHD diagnosis	Same as for specification<br>Required data for each person: age of first diagnosis
Intervention assignment	How will eligible persons be assigned to the interventions	Eligible children will be randomly assigned to one intervention and will be aware of it	Eligible children will be assigned to the intervention with which their data are compatible at the time of eligibility and will be aware of it
Outcomes	What outcomes in eligible persons will be compared among intervention groups?	9 pre-determined, self-reported measures of quality of life	Same as for specification<br>Required data: values for outcome measures at end of study
Follow-up	During which period will eligible persons be followed in the study?	From intervention assignment until loss to follow-up or administrative end of follow-up at age 14/15	Same as for specification<br>Required data: N/A (due to multiple imputations used to estimate data)
Causal estimand	Which counterfactual contrasts will be estimated?	Intention-to-treat effect (effect of being assigned an ADHD diagnosis)	Observational analogue of intention-to-treat effect
Statistical analysis	How will counterfactual contrasts be estimated?	Comparing quality of life outcomes at age 14/15 between an equal number of children randomly assigned to each intervention strategy by calculating mean differences/odds ratios for each outcome; regardless of adherence for intention-to-treat analysis.	Same as specification<br>Required data: pre-assignment confounders

[a]following the approach described in
Hernan MA. Methods of Public Health Research — Strengthening Causal Inference from Observational Data. New England Journal of Medicine. 2021;385(15).

© 2022 Kazda L et al. *JAMA Network Open.*

eTable 2. Outcome Measures

Outcome measure	WHO quality of life domain	Response format	Question(s)	Source
Academic self-concept	Environment & Psychological	Total score: 1-4<br>(Average of 3Qs with 1=Strongly disagree, 2=Disagree, 3=Agree, 4=Strongly agree)<br>Higher scores indicate a more positive academic self-concept.	1. I do well in tests in most school subjects<br>2. I learn things quickly in most school subjects<br>3. I'm good at most school subjects	Adapted from the Program for International Student Assessment (PISA) (2000).
Child Health Utility 9D	All 4 dimensions	Total score: 0-1<br>(Calculated from the CHU9D items using a weighted algorithm from a sample of Australian adolescents)<br>Higher scores indicate better health related quality of life.	These questions ask about how you are today. For each question, read all the choices and decide which one is most like you today<br>1. Worried: I don't feel worried today, I feel a little bit worried today, I feel a bit worried today, I feel quite worried today, I feel very worried today<br>2. Sad: I don't feel sad today, I feel a little bit sad today, I feel a bit sad today, I feel quite sad today, I feel very sad today<br>3. Pain: I don't have any pain today, I have a little bit of pain today, I have a bit of pain today, I have quite a lot of pain today, I have a lot of pain today<br>4. Tired: I don't feel tired today, I feel a little bit tired today, I feel a bit tired today, I feel quite tired today, I feel very tired today<br>5. Annoyed: I don't feel annoyed today, I feel a little bit annoyed today, I feel a bit annoyed today, I feel quite annoyed today, I feel very annoyed today<br>6. Work/study (Things like paid work, voluntary work, housework and study): I have no problems with my schoolwork/homework today, I have a few problems with my schoolwork/homework today, I have some problems with my schoolwork/homework today, I have many problems with my schoolwork/homework today, I can't do my schoolwork/homework today<br>7. Sleep: Last night I had no problems sleeping, Last night I had a few problems sleeping, Last night I had some problems sleeping, Last night I had many problems sleeping, Last night I couldn't sleep at all<br>8. Daily routine (Things like eating, having a bath/shower, getting dressed): I have no problems with my daily routine today, I have a few problems with my daily routine today, I have some problems with my daily routine today, I have many problems with my daily routine today, I can't do my daily routine today<br>9. Able to join in activities (Things like playing out with your friends, doing sports, joining in things): I can join in with any activities today, I can join in with most activities today, I can join in with some activities	Adapted from the Child Health Utility 9D (CHU9D) (Stevens, 2005).

© 2022 Kazda L et al. *JAMA Network Open.*

			today, I can join in with a few activities today, I can join in with no activities today	
Global health	Physical	Total score: 1-5<br>(1=Excellent, 2=Very good, 3=Good, 4=Fair, 5=Poor)<br>Lower scores indicate better health perception	How would you rate your general health?	Adapted from the Medical Outcomes Study (MOS) 36-Item Short-Form Health Survey (SF-36) (Ware & Sherbourne, 1992).
Negative social behaviours	Social relationships	Total score: 0-85<br>(Total of 17 Qs with 0=Not at all, 1=Once, 2=Twice, 3= Three times, 4=Four times, 5=Five or more)<br>Lower scores indicate less negative social behaviour occurrences	In the last 12 months had you…?<br>1. Been caught by police for something you had done<br>2. Been suspended or expelled from school,<br>3. Broken into a house, flat or vehicle<br>4. Carried a weapon like a knife, gun or piece of wood<br>5. Damaged a parked car (e.g. broken an aerial, slashed tyres, scratched paint)<br>6. Drawn graffiti in public places<br>7. Gone around with a group of 3 or more kids damaging property or getting into fights<br>8. Got into physical fights in public<br>9. Purposely damaged or destroyed others' property<br>10. Run away from home and stayed away overnight or longer<br>11. Skipped school for a whole day<br>12. Started a fire in a place where you should not burn anything<br>13. Stolen money or other things from another person<br>14. Stolen something from a shop<br>15. Stolen something out of a parked car<br>16. Taken a vehicle (e.g. car, motorbike) for a ride or drive without permission<br>17. Used force or threats to get money or things from someone	Adapted from Self-Report Delinquency Scale developed by Moffit and Silva (1988).

© 2022 Kazda L et al. *JAMA Network Open.*

Overall happiness	Psychological	Total score: 1-5<br>(1=Strongly disagree, 2=Disagree, 3=Neither, 4=Agree, 5= Strongly agree)<br>Higher scores indicate more happiness	In general, I am happy with how things are for me in my life right now.	Drawn from the Western Australian Child Development Study, Youth Questionnaire, 12-17 years, Telethon Institute for Child Health Research.
Peer trust	Social relationships	Total score: 4-20<br>(Total of 4Qs with 1=Almost always true, 2=Often true, 3=Sometimes true, 4=Seldom true, 5=Almost never true)<br>Lower scores indicate better peer trust	1. I feel my friends are good friends<br>2. My friends listen to what I say<br>3. I trust my friends<br>4. My friends respect my feelings	Adapted from the Trust subscale of the Peer Attachment Scale, Armsden and Greenberg (from the Inventory of Peer and Parental Attachment (1987)).
Psychological sense of school membership	Social relationships	Total score: 12-60<br>(Total of 12Qs with 1=Completely true, 2= Somewhat true, 3=Neither, 4=Not very true, 5=Not at all true; some reverse coded)<br>Higher scores indicate better psychological sense of school membership	For each of the following sentences please pick the answer that best matches your experience. There are no right or wrong answers.<br>1. I am included in lots of activities at this school.<br>2. I can really be myself at this school.<br>3. I wish I were in a different school.<br>4. It is hard for people like me to be accepted here.<br>5. Most teachers at this school are interested in me.<br>6. Other students here like me the way I am.<br>7. Other students in this school take my opinions seriously.<br>8. People here notice when I'm good at something.<br>9. Sometimes I don't feel as if I belong here.<br>10. Teachers here are not interested in people like me.<br>11. The teachers here respect me.<br>12. There's at least one teacher or other adult in this school I can talk to if I have a problem.	Adapted from the Psychological Sense of School Membership Scale (PSSM), Goodenow, 1993.
Self-efficacy	Psychological	Total score: 1-5<br>(Average of 5Qs with 1=False, 2=Mostly False, 3=Sometimes false sometimes true, 4=Mostly true, 5=True)<br>Higher scores indicate better self-efficacy	1. I can do things as well as most people<br>2. If I really try I can do almost anything I want<br>3. Most things I do, I do well<br>4. Overall, I have a lot to be proud of<br>5. Overall, most things I do turn out well	Adapted from Marsh Self Description Questionnaire II (Marsh, 1990).
Self-harm	Psychological	Total score: 0 or1<br>(1=Yes, 0=No)	During the past 12 months have you hurt yourself on purpose in any way?	LSAC designed item.

© 2022 Kazda L et al. *JAMA Network Open.*

eTable 3. Propensity Score Balance Assessment[a]

	Age 6/7		Age 8/9		Age 10/11		Age 12/13		Age 14/15	
	**SMD[b]**	**Variance ratio**	**SMD[b]**	**Variance ratio**	**SMD[b]**	**Variance ratio**	**SMD[b]**	**Variance ratio**	**SMD[b]**	**Variance ratio**
Overall propensity score	0.01	1.11	0.01	1.08	0.02	1.05	0.01	1.12	0.00	1.02
Cohort	0.03	1.00	0.03	0.99	0.02	1.01	0.02	1.02	0.02	1.02
Sex	0.00	1.00	0.00	1.00	0.00	1.00	0.00	1.00	0.00	1.00
Aboriginal and/or Torres Strait Islander	0.04	1.20	0.08	1.49	0.04	1.66	0.04	1.25	0.01	0.80
Main language spoken at home	0.03	0.91	0.01	1.03	0.00	1.15	0.04	0.93	0.00	1.28
Two parent household	0.00	1.03	0.03	0.97	0.01	1.07	0.02	1.14	0.07	1.55
Number of siblings	0.03	1.33	0.02	1.00	0.02	1.00	0.00	1.02	0.09	0.83
Number of stressful life events	0.00	1.11	0.03	0.98	0.01	1.07	0.00	0.64	0.01	0.85
Rurality	0.02	1.03	0.01	1.00	0.01	1.02	0.01	1.01	0.03	1.00
SEIFA Advantage/ Disadvantage score	0.01	0.94	0.03	0.99	0.00	1.22	0.02	0.87	0.10	1.26
Socioeconomic position	0.01	1.54	0.01	0.99	0.01	0.99	0.03	0.90	0.05	1.54
Mother's school completion	0.00	1.01	0.01	1.01	0.02	1.02	0.05	1.06	0.02	1.01
Mother's further education	0.01	1.12	0.01	0.98	0.00	0.79	0.01	0.96	0.05	0.95
Comorbidities	0.00	0.79	0.01	0.87	0.08	0.76	0.06	1.05	0.03	1.21
Depression/ anxiety	N/A	N/A	N/A	N/A	N/A	N/A	0.04	1.15	0.14	1.44
Autism	N/A	N/A	N/A	N/A	N/A	N/A	0.15	1.40	0.06	0.96
N of other comorbidities	0.00	0.79	0.01	0.87	0.08	0.76	0.06	1.05	0.03	1.21
SDQ H/I score	0.02	1.06	0.01	0.96	0.00	0.99	0.03	1.11	0.03	0.79

[a]average from 25 multiple imputation sets
[b]Standardised mean difference

© 2022 Kazda L et al. *JAMA Network Open.*

eTable 4. Missing Data

	Age 4/5 (n=8503)			Age 6/7 (n=8643)			Age 8/9 (n=8093)			Age 10/11 (n=7625)			Age 12/13 (n=7070)			Age 14/15 (n=6433)		
**Variable**	Missing values	Non-participants	% of data imputed	Missing values	Attrition	% of data imputed	Missing values	Attrition	% of data imputed	Missing values	Attrition	% of data imputed	Missing values	Attrition	% of data imputed	Missing values	Attrition	% of data imputed
Sex[a]	0	0	0.0	0	0	0.0	0	0	0.0	0	0	0.0	0	0	0.0	0	0	0.0
Aboriginal/Torres Strait Islander status[a]	0	0	0.0	0	0	0.0	0	0	0.0	0	0	0.0	0	0	0.0	0	0	0.0
Cohort[a]	0	0	0.0	0	0	0.0	0	0	0.0	0	0	0.0	0	0	0.0	0	0	0.0
Language spoken at home[a]	2	0	0.0	2	0	0.0	1	0	0.0	1	0	0.0	1	0	0.0	0	0	0.0
Socio-economic position[b]	14	135	1.7	4	0	0.0	56	550	7.0	76	1018	12.7	118	1573	19.6	100	2210	26.7
SEIFA adv/disadv score[b]	0	135	1.6	0	0	0.0	7	550	6.4	1	1018	11.8	5	1573	18.3	1	2210	25.6
Number of siblings[b]	0	135	1.6	0	0	0.0	7	550	6.4	7	1018	11.9	62	1573	18.9	14	2210	25.7
Two-parent household[b]	0	135	1.6	0	0	0.0	7	550	6.4	7	1018	11.9	62	1573	18.9	14	2210	25.7
H/I SDQ score[b]	478	135	7.1	150	0	1.7	561	550	12.9	134	1018	13.3	134	1573	19.8	189	2210	27.8
Stressful life events[b]	1161	135	15.0	65	0	0.8	702	550	14.5	139	1018	13.4	142	1573	19.8	195	2210	27.8
State of residency[b]	0	135	1.6	0	0	0.0	0	550	6.4	0	1018	11.8	1	1573	18.2	0	2210	25.6
Rurality[b]	11	135	1.7	0	0	0.0	7	550	6.4	15	1018	12.0	1	1573	18.2	0	2210	25.6
Mother's schooling[b]	44	135	2.1	21	0	0.2	83	550	7.3	91	1018	12.8	161	1573	20.1	136	2210	27.1
Mother's further education[b]	292	135	4.9	25	0	0.3	66	550	7.1	77	1018	12.7	123	1573	19.6	119	2210	26.9
Other comorbidities[b]	0	135	1.6	3	0	0.0	31	550	6.7	66	1018	12.5	116	1573	19.5	93	2210	26.6
ADHD diagnosis	0	135	1.6	3	0	0.0	31	550	6.7	66	1018	12.5	116	1573	19.5	93	2210	26.6
Depression/Anxiety[b]										66	1018	12.5	116	1573	19.5	93	2210	26.6
Autism[b]										66	1018	12.5	116	1573	19.5	93	2210	26.6
Overall happiness[c]													284	1573	21.5	303	2210	29.1
Self-efficacy[c]													282	1573	21.5	300	2210	29.0
Negative social behaviours[c]													282	1573	21.5	301	2210	29.1
Psychological sense of school membership[c]													441	1573	23.3	364	2210	29.8
Peer trust[c]													275	1573	21.4	302	2210	29.1

© 2022 Kazda L et al. *JAMA Network Open.*

Academic self-concept																304	2210	29.1
Child Health Utility 9D																323	2210	29.3
Self-harm																357	2210	29.7
Global health																313	2210	29.2

[a]variables were treated as fixed and values at age 6/7 were used for all ages

[b]values at age 14/15 were only used as auxiliary variable to impute values at previous ages, not for analysis model

[c]values at age 12/13 were only used as auxiliary variables to impute outcome values at age 14/15, not for analysis model

© 2022 Kazda L et al. *JAMA Network Open.*

eTable 5. Number of Participants in Exposure (Drop-outs) and Control (Drop-ins) Groups with an ADHD Diagnosis During Follow-up[a]

	H/I SDQ score[b]	n	Has diagnosis at age				
			6/7	8/9	10/11	12/13	14/15
**First dx at 6/7**	Low risk	29	100.0% (29)	65.5% (19)	58.6% (17)	48.3% (14)	55.2% (16)
	Borderline	40	100.0% (40)	80.0% (32)	70.0% (28)	70.0% (28)	82.5% (33)
	High risk	22	100.0% (22)	77.3% (17)	77.3% (17)	77.3% (17)	72.7% (16)
	**Total**	**91**	**100.0% (91)**	**74.7% (68)**	**68.1% (62)**	**64.8% (59)**	**71.4% (65)**
**No diagnosis at 6/7**	Low risk	26	0.0% (0)	0.0% (0)	0.0% (0)	0.0% (0)	0.0% (0)
	Borderline	44	0.0% (0)	4.5% (2)	2.3% (1)	0.0% (0)	0.0% (0)
	High risk	21	0.0% (0)	4.8% (1)	4.8% (1)	0.0% (0)	0.0% (0)
	**Total**	**91**	**0.0% (0)**	**3.3% (3)**	**2.2% (2)**	**0.0% (0)**	**0.0% (0)**
**First dx at 8/9**	Low risk	41		100.0% (41)	68.3% (28)	61.0% (25)	51.2% (21)
	Borderline	61		100.0% (61)	73.8% (45)	67.2% (41)	67.2% (41)
	High risk	23		100.0% (23)	69.6% (16)	73.9% (17)	69.6% (16)
	**Total**	**125**		**100.0% (125)**	**71.2% (89)**	**66.4% (83)**	**62.4% (78)**
**No diagnosis at 8/9**	Low risk	49		0.0% (0)	4.1% (2)	2.0% (1)	2.0% (1)
	Borderline	55		0.0% (0)	7.3% (4)	1.8% (1)	0.0% (0)
	High risk	21		0.0% (0)	4.8% (1)	4.8% (1)	0.0% (0)
	**Total**	**125**		**0.0% (0)**	**5.6% (7)**	**6.4% (3)**	**0.8% (1)**
**First dx at 10/11**	Low risk	30			100.0% (30)	83.3% (25)	73.3% (22)
	Borderline	37			100.0% (37)	75.7% (28)	67.6% (25)
	High risk	12			100.0% (12)	75.0% (9)	75.0% (9)
	**Total**	**79**			**100.0% (79)**	**78.5% (62)**	**70.9% (56)**
**No diagnosis at 10/11**	Low risk	36			0.0% (0)	0.0% (0)	0.0% (0)
	Borderline	31			0.0% (0)	3.2% (1)	0.0% (0)
	High risk	13			0.0% (0)	7.7% (1)	0.0% (0)
	**Total**	**80**			**0.0% (0)**	**2.5% (2)**	**0.0% (0)**
**First dx at 12/13**	Low risk	27				100.0% (27)	51.9% (14)
	Borderline	21				100.0% (21)	66.7% (14)
	High risk	8				100.0% (8)	62.5% (5)
	**Total**	**56**				**100.0% (56)**	**58.9% (33)**
**No diagnosis at 12/13**	Low risk	29				0.0% (0)	0.0% (0)
	Borderline	20				0.0% (0)	0.0% (0)
	High risk	6				0.0% (0)	0.0% (0)
	**Total**	**55**				**0.0% (0)**	**0.0% (0)**
**First dx at 14/15**	Low risk	26					100.0% (26)
	Borderline	11					100.0% (11)
	High risk	4					100.0% (4)
	**Total**	**41**					**100.0% (41)**
**No diagnosis at 14/15**	Low risk	23					0.0% (0)
	Borderline	14					0.0% (0)
	High risk	4					0.0% (0)
	**Total**	**41**					**0.0% (0)**

© 2022 Kazda L et al. *JAMA Network Open.*

**Total diagnosis group**	Low risk	153					64.7% (99)
	Borderline	170					72.9% (124)
	High risk	69					72.5% (50)
	**Total**	**392**					**69.6% (273)**
**Total no diagnosis group**	Low risk	163					2.5% (4)
	Borderline	164					5.5% (9)
	High risk	65					7.7% (5)
	**Total**	**392**					**4.6% (18)**

[a]average from 25 imputed datasets

[b]Strengths and Difficulties Questionnaire Hyperactivity/Inattention Subscale scores: Low risk: 0-4 points, Borderline=5-7points, High risk=8-10 points

© 2022 Kazda L et al. *JAMA Network Open.*

eTable 6. Association of ADHD Diagnosis With Quality-of-Life Outcomes at Age 14/15 Years: Complete Case Analysiss

Outcome	w/out ADHD diagnosis (n=238), mean	with ADHD diagnosis (n=238), mean	Mean difference	95% CI		p-value	Effect size (Cohen's d)[a]	Interpretation
				Lower	Higher			
**Child health utility 9D** (range 0-1, higher scores indicate better QoL)	0.83	0.80	-0.03	-0.06	0.01	0.128	0.13	There is no difference in CHU9D scores.
**Academic self-concept** (range 1-4, higher scores indicate a more positive academic self-concept)	2.92	2.73	-0.19	-0.31	-0.07	**0.008**	**0.30**	Teens with diagnosis have a worse academic self-concept than those without diagnosis.
**Global health** (range 1-5, lower scores indicate better health perception)	2.19	2.24	0.05	-0.10	0.22	0.464	0.06	There is no difference in perceived health.
**Negative social behaviours** (range 0-85, lower scores indicate less negative social behaviours)	1.58	2.83	1.25	0.47	2.17	**0.008**	**0.26**	Teens with diagnosis display more negative social behaviours than those without diagnosis.
**Overall happiness** (range 1-5, higher scores indicate more happiness)	3.87	3.74	-0.13	-0.32	0.08	0.232	0.11	There is no difference in overall happiness
**Peer trust** (range 4-20, lower scores indicate better peer trust)	9.24	10.10	0.86	0.15	1.61	**0.012**	**0.22**	Teens with diagnosis have lower trust in their peers than those without diagnosis.[b]
**Psychological sense of school membership** (range 12-60, higher scores indicate better pssm)	48.03	45.26	-2.77	-4.34	-1.23	**<0.001**	**0.33**	Teens with diagnosis have a worse psychological sense of school membership than those without diagnosis.
**Self-efficacy** (range 1-5, higher scores indicate better self-efficacy)	3.93	3.75	-0.17	-0.32	-0.03	**0.012**	**0.23**	Teens with diagnosis have less self-efficacy than those without diagnosis.
**Self-harm, % and OR** (yes)	7.1	13.5	2.03	1.07	4.37	**0.036**	N/A	Teens with diagnosis are more than twice as likely to harm themselves than those without diagnosis

[a]Cohen's d:
<0.20: negligible effect, 0.20-0.49: small effect,
0.50-0.79: moderate effect, >=0.80: large effect
[b]This result differs to main analysis where there was no significant difference.

© 2022 Kazda L et al. *JAMA Network Open.*

eTable 7. Association of ADHD Diagnosis With Quality of Life by H/I SDQ Score[a,b]

Outcome	Low risk with ADHD diagnosis (n=163), mean	Low risk w/out ADHD diagnosis (n=153), mean	Mean difference	95% CI		p-value	Borderline with ADHD diagnosis (n=164), mean	Borderline w/out ADHD diagnosis (n=170), mean	Mean difference	95% CI		p-value	High risk with ADHD diagnosis (n=66), mean	High risk w/out ADHD diagnosis (n=69), mean	Mean difference	95% CI		p-value
				Lower	Higher					Lower	Higher					Lower	Higher	
**Child health utility 9D** (range 0-1, higher scores indicate better QoL)	0.77	0.81	-0.04	-0.10	0.04	0.309	0.77	0.80	-0.04	-0.09	0.02	0.171	0.78	0.80	-0.01	-0.08	0.05	0.687
**Academic self-concept** (range 1-4, higher scores indicate a more positive academic self-concept)	2.71	2.92	-0.21	-0.47	0.04	0.105	2.65	2.84	**-0.19**	-0.35	-0.01	**0.042**	2.75	2.78	-0.03	-0.23	0.17	0.733
**Global health** (range 1-5, lower scores indicate better health perception)	2.17	2.06	0.11	-0.22	0.42	0.495	2.38	2.19	0.19	-0.03	0.43	0.092	2.28	2.27	0.01	-0.28	0.30	0.967
**Negative social behaviours** (range 0-85, lower scores indicate less negative social behaviours)	3.82	1.49	**2.32**	0.05	4.57	**0.044**	3.51	1.89	**1.62**	0.21	3.14	**0.024**	3.47	2.57	0.91	-0.86	2.87	0.301
**Overall happiness** (range 1-5, higher scores indicate more happiness)	3.76	3.84	-0.08	-0.48	0.38	0.713	3.48	3.74	-0.27	-0.60	0.04	0.091	3.59	3.75	-0.16	-0.48	0.18	0.368
**Peer trust** (range 4-20, lower scores indicate better peer trust)	10.07	9.37	0.70	-0.66	1.87	0.305	10.52	9.58	0.94	-0.08	1.93	0.071	10.09	9.86	0.23	-1.03	1.56	0.764

© 2022 Kazda L et al. *JAMA Network Open.*

**Psychological sense of school membership** (range 12-60, higher scores indicate better pssm)	45.23	48.13	-2.89	-6.03	0.61	0.101	43.37	47.03	**-3.66**	-6.17	-1.34	**0.001**	44.76	45.67	-0.91	-3.28	1.68	0.475
**Self-efficacy** (range 1-5, higher scores indicate better self-efficacy)	3.66	3.96	**-0.30**	-0.56	-0.02	**0.036**	3.58	3.86	**-0.28**	-0.49	-0.08	**0.005**	3.75	3.75	-0.01	-0.25	0.25	0.923
**Self-harm, % and OR** (yes)	20.6	7.5	**3.44**	1.14	19.80	**0.027**	21.5	9.2	**2.81**	1.25	7.65	**0.010**	18.0	10.5	**2.65**	1.53	5.41	**0.001**

[a]Strengths and Difficulties Questionnaire Hyperactivity/Inattention Subscale scores: Low risk: 0-4 points, Borderline=5-7points, High risk=8-10 points

[b]Shading: green=smallest difference, orange=middle difference, red= biggest difference

© 2022 Kazda L et al. *JAMA Network Open.*

eTable 8. Association of ADHD Diagnosis With Quality of Life by Sex[a]

Outcome	Boys with ADHD diagnosis (n=284), mean	Boys w/out ADHD diagnosis (n=284), mean	Mean difference	95% CI		p-value	Girls with ADHD diagnosis (n=109), mean	Girls w/out ADHD diagnosis (n=109), mean	Mean difference	95% CI		p-value
				Lower	Higher					Lower	Higher	
**Child health utility 9D** (range 0-1, higher scores indicate better QoL)	0.81	0.83	-0.02	-0.06	0.02	0.316	0.68	0.73	-0.05	-0.13	0.03	0.227
**Academic self-concept** (range 1-4, higher scores indicate a more positive academic self-concept)	2.75	2.86	-0.11	-0.23	0.02	0.100	2.56	2.79	-0.23	-0.45	0.00	0.051
**Global health** (range 1-5, lower scores indicate better health perception)	2.22	2.14	0.08	-0.10	0.26	0.381	2.49	2.30	0.19	-0.11	0.49	0.197
**Negative social behaviours** (range 0-85, lower scores indicate less negative social behaviours)	3.84	2.12	**1.72**	0.53	2.92	**0.004**	2.89	1.74	1.15	-0.80	3.23	0.230
**Overall happiness** (range 1-5, higher scores indicate more happiness)	3.66	3.82	-0.16	-0.36	0.05	0.146	3.38	3.64	-0.26	-0.62	0.10	0.164
**Peer trust** (range 4-20, lower scores indicate better peer trust)	10.75	10.10	0.65	-0.13	1.41	0.100	9.01	8.36	0.66	-0.62	1.91	0.324
**Psychological sense of school membership** (range 12-60, higher scores indicate better pssm)	44.79	47.37	**-2.58**	-4.33	-0.90	**0.002**	42.95	45.51	-2.56	-5.33	0.54	0.102
**Self-efficacy** (range 1-5, higher scores indicate better self-efficacy)	3.68	3.88	**-0.21**	-0.36	-0.05	**0.011**	3.60	3.77	-0.17	-0.47	0.12	0.260
**Self-harm, % and OR** (yes)	16.9	6.2	**2.53**	1.45	4.74	**<0.001**	28.5	17.1	2.00	0.87	5.21	0.100

[a]Shading: green=smallest difference, red= biggest difference

© 2022 Kazda L et al. *JAMA Network Open.*

eTable 9. Association of ADHD Diagnosis With Quality of Life at Age 14/15 Years by Age at First Diagnosis (Time Zero)[a]

Outcome	Age 6/7 with ADHD diagnosis (n=91), mean	Age 6/7 w/out ADHD diagnosis (n=91), mean	Mean difference	95% CI		p-value	Age 10/11 ADHD diagnosis (n=80), mean	Age 10/11 w/out ADHD diagnosis (n=80), mean	Mean difference	95% CI		p-value	Age 14/15 with ADHD diagnosis (n=41), mean	Age 14/15 w/out ADHD diagnosis (n=41), mean	Mean difference	95% CI		p-value
				Lower	Higher					Lower	Higher					Lower	Higher	
**Child health utility 9D** (range 0-1, higher scores indicate better QoL)	0.76	0.80	-0.04	-0.12	0.04	0.375	0.81	0.82	-0.01	-0.08	0.06	0.796	0.76	0.78	-0.02	-0.13	0.08	0.646
**Academic self-concept** (range 1-4, higher scores indicate a more positive academic self-concept)	2.66	2.84	-0.18	-0.42	0.06	0.157	2.78	2.82	-0.03	-0.28	0.23	0.787	2.50	2.84	-0.33	-0.67	0.01	0.057
**Global health** (range 1-5, lower scores indicate better health perception)	2.37	2.16	0.21	-0.12	0.54	0.224	2.31	2.17	0.14	-0.20	0.46	0.409	2.27	2.15	0.12	-0.37	0.60	0.611
**Negative social behaviours** (range 0-85, lower scores indicate less negative social behaviours)	4.66	1.93	**2.73**	0.26	5.38	**0.032**	2.95	1.85	1.10	-0.84	3.11	0.253	3.33	1.95	1.39	-1.39	3.73	0.257
**Overall happiness** (range 1-5, higher scores indicate more happiness)	3.33	3.76	-0.43	-0.81	0.01	0.056	3.72	3.74	-0.03	-0.43	0.38	0.886	3.82	3.76	0.06	-0.43	0.62	0.833
**Peer trust** (range 4-20, lower scores indicate	10.73	9.55	1.18	-0.31	2.65	0.119	10.11	9.62	0.49	-1.09	1.96	0.544	9.18	9.26	-0.08	-1.93	1.68	0.936

© 2022 Kazda L et al. *JAMA Network Open.*

better peer trust)																	
**Psychological sense of school membership** (range 12-60, higher scores indicate better pssm)	42.10	46.87	**-4.78**	-7.96	-1.53	**0.003**	46.36	46.88	-0.52	-3.58	2.54	0.740	43.41	47.52	-4.11	-7.93	0.05	0.053
**Self-efficacy** (range 1-5, higher scores indicate better self-efficacy)	3.56	3.87	-0.32	-0.60	0.00	0.052	3.66	3.86	-0.20	-0.49	0.08	0.161	3.70	3.84	-0.15	-0.52	0.23	0.461
**Self-harm, % ad OR** (yes)	29.5	9.5	**2.22**	1.58	15.15	**0.004**	18.5	6.8	**1.78**	1.00	28.11	**0.048**	17.2	12.5	**1.30**	1.37	39.93	**0.003**

[a]Shading: green=smallest difference, orange=middle difference, red= biggest difference

© 2022 Kazda L et al. *JAMA Network Open.*
